# Simple Sequence Repeat Polymorphisms (SSRPs) for Evaluation of Molecular Diversity and Germplasm Classification of Minor Crops

**DOI:** 10.3390/molecules14114546

**Published:** 2009-11-10

**Authors:** Yong-Jin Park, Ju Kyong Lee, Nam-Soo Kim

**Affiliations:** 1 Department of Plant Sciences, Kongju National University, Yesan 340-702, Korea; 2 Division of Bio-Resources Technology, Kangwon National University, Chunchon 200-701, Korea; 3 Department of Molecular Biosciences, Kangwon National University, Chunchon 200-701, Korea; 4 Institute of Biosciences and Biotechnology, Kangwon National University, Chunchon 200-701, Korea

**Keywords:** genetic diversity, germplasm, DNA polymorphism, molecular markers, SSR

## Abstract

Evaluation of the genetic diversity among populations is an essential prerequisite for the preservation of endangered species. Thousands of new accessions are introduced into germplasm institutes each year, thereby necessitating assessment of their molecular diversity before elimination of the redundant genotypes. Of the protocols that facilitate the assessment of molecular diversity, SSRPs (simple sequence repeat polymorphisms) or microsatellite variation is the preferred system since it detects a large number of DNA polymorphisms with relatively simple technical complexity. The paucity of information on DNA sequences has limited their widespread utilization in the assessment of genetic diversity of minor or neglected crop species. However, recent advancements in DNA sequencing and PCR technologies in conjunction with sophisticated computer software have facilitated the development of SSRP markers in minor crops. This review examines the development and molecular nature of SSR markers, and their utilization in many aspects of plant genetics and ecology.

## Introduction

Since the onset of life on earth more than three billion years ago, Mother Nature has generated a plethora of diverse life forms. Although genetic diversity is the most important legacy that one generation can pass on to the next, numerous species have disappeared over the years, and will continue to be lost. For example, it has been suggested that the current climate change may threaten the survival of as much as 12% of the wild relatives of Solanum (http://www.potato2008.org/en/ potato/biodiversity.html). Although modern agricultural practices, urban expansion, deforestation, and other human activities all contribute to the pace with which species are becoming endangered and extinct, significant international efforts have been implemented to impede the erosion of genetic diversity. More than 600,000 plant samples are held by CGIAR (Consultative Group on International Research) to preserve the biodiversity of crop species (http://ftp.fao.org/planttreaty/news/ news0003_2n.pdf), and hundreds of thousands of new samples are introduced into the germplasm institutes each year. However, it is necessary to assess the genetic variations in these introductions for redundancy. In an effort to maximize genetic variability and minimize repetitiveness, the concept of ‘core collection’ was introduced to maintain the optimum size of samples in a population [1,2,3].

Genetic diversity is influenced by selection, mutation, migration, population size, and genetic drift [4,5], and understanding how each of these factors influences the genetic diversity of a population is critical to the conservation of species. Although morphological markers enable the detection of genetic variation, it is often disguised by factors in the environment, and minimized by a paucity of discernible morphological markers. Pleiotropism and the late onset of some morphological markers during plant development also render unequivocal assessment difficult [6]. Significant advancements in molecular biology have shifted the focus of assessment of biodiversity from relying on morphological markers to using isozymes and DNA markers [7,8,9]. This review will focus on Simple Sequence Repeat Polymorphisms (SSRPs) as a molecular marker system for the assessment of genetic diversity in plants, with particular emphasis on the neglected minor crops or endangered species.

## Molecular Genetic Markers in Plants

Variation between individuals in a population or between populations in a species, derived from genes and/or environmental effects, can be easily evaluated through the use of a variety of markers. Genetic markers were described before the discovery of proteins and DNA. In 1913, Alfred Sturtevant mapped six morphological markers that he described as “factors” on the chromosomes of the fruit fly [10]. Karl Sax demonstrated that seed size differences, seed-coat and pigmentation patterns were genetically linked in the common bean, *Phaseolus vulgaris* [11], where the number of easily discernible morphological markers was limited. Morphological traits that exhibit continuous variation between individuals in a population often obscure the evaluation of genetic diversity. Moreover, pleiotropism and a multifactorial basis to morphological traits further obfuscate the characterization of plant populations. The discovery that genes encoded proteins and enzymes led to the utilization of isozymes and other proteins as marker systems for genetic analysis of populations [7,12,13]. Although protein markers circumvent the effects of environment, they have the drawbacks of a limitation in the number of detectable isozymes as well as tissue and development stage specificity.

DNA marker systems, which were introduced to genetic analysis in the 1980s, have many advantages over the traditional morphological and protein markers that are used in genetic and ecological analyses of plant populations: firstly, an unlimited number of DNA markers can be generated; secondly, DNA marker profiles for are not affected by the environment, and, thirdly DNA markers, unlike isozyme markers, are not constrained by tissue or developmental stage specificity.

The first generation of DNA marker systems employed Southern blot based markers. RFLPs (restriction fragment length polymorphisms) result from point mutations in restriction enzyme recognition sites. Chromosomal mutations such as insertions, deletions, inversions, and translocations can also cause restriction fragment size polymorphisms. The RFLP technique employs molecular hybridization of cDNA or genomic DNA probes with genomic DNA fragmented by restriction enzymes. Subsequent to the first demonstration of the usefulness of RFLPs in human genetics for linkage analysis [14], the technique was soon adopted by plant research communities [15,16,17]. Another Southern blot based marker system relied on minisatellite probes for ‘fingerprinting’ individual specific human DNA [18]. Minisatellite DNAs are short stretches of DNA that are present in tandem repeats in eukaryotes. They are highly abundant, and individuals often carry different numbers of tandem repeats which can be detected as VNTRs (variable number of tandem repeat) by PCR amplification [19]. While the RFLP technique utilizes mostly low copy number probes, the fingerprinting technique uses highly repetitive minisatellite DNAs as probes. Plant genomes also harbor many families of minisatellites [20,21,22,23]. Human minisatellite probes have been successfully used to distinguish individual Gramineae plants [24]. In modestly equipped laboratories, the technical complexity and high cost of these Southern blot based marker systems often limits their utilization in genetic analyses of large populations.

The second generation DNA markers for genetic analysis were those derived from PCR polymerase chain reaction [25]. PCR revolutionized genetic and ecological analyses of populations in several ways because it had two major advantages over Southern blot based markers. First, it requires only small DNA amounts to allow analysis at very early stages, thus reducing the need for plant nurseries. Second, it is inexpensive, and simple enough that large scale experiments can be carried out rapidly on a large scale. Of the many PCR-marker techniques that have been developed, RAPD, AFLP and SSR are the major systems, with the other systems being modifications of these three. The RAPD (randomly amplified polymorphic DNA) system may be the most efficient and simplest of techniques among the molecular genetic marker systems [26,27,28]. The RAPD technique utilizes a single arbitrary primer of 10-12 nucleotides (usually 10 nucleotides long) in the PCR reaction and thus, does not require template DNA sequence information. RAPD primers can bind complementary sequences in the genome and amplify the target sequences to produce 1-10 amplicons, depending on the templates and primers, and if the primer binding sites are within amplifying distance (100–1,000 bp). Although widely used in many analyses, the simple RAPD protocol has the significant drawback of a low reproducibility of results [29]. The dominant nature of RAPD markers also limits their application in F2 population and parentage analysis. However, these limitations can be overcome by converting RAPD markers to STS (sequence-tag-site) markers [30,31,32]. Inter-SSR is a modified version of the RAPD technique with SSR (simple sequence repeat) strategy, and is explained in reviews elsewhere [33,34]. Inter-SSR uses primers consisting of SSR sequences and amplifies template DNA as does RAPD, but its target sequences are mostly in regions of the genome that harbor SSR sites. Low reproducibility of results with inter-SSR markers also poses as an impediment to its widespread use.

AFLP (amplified fragment length polymorphism), another PCR-based molecular marker system, obviates the need for template DNA sequence information [35]. Genomic DNA digested with two different restriction enzymes is ligated with specific adaptor sequences. The adaptor-ligated restriction fragments are then amplified with adaptor complementary primers that have selective nucleotides at their 3’-ends. The amplified fragments are separated by denaturing polyacrylamide gel electrophoresis and then stained with silver. Reproducibility of AFLP profiles is reasonably high, and each AFLP reaction produces 40-50 anonymous amplicons, ranging from 100–600 bp. The reproducibility of AFLPs was tested throughout a network of European laboratories in which AFLP banding patterns were reproduced in a range of laboratories by rigorous control of all the variables [29]. Partial digestion of the template DNAs by contamination or organ specific methylation produces spurious AFLP bands [36,37]. The anonymous multi-bands render AFLPs a preferred protocol for fingerprinting purposes [35,38]. AFLP markers exhibit dominant inheritance which can be converted to co-dominant STS markers to detect alleles of a given locus [29]. Another popular DNA marker system is the SSR, and since this review focuses on SSRPs, a detailed discussion of SSRs will follow at the end of this section. 

The third generation of molecular markers is the system that utilizes SNPs (single nucleotide polymorphisms) [39,40,41] and microarrays [42]. Compared to the gel-based molecular marker systems, SNP detection and analysis can be carried out with non-gel based assays. The polymorphism of a single base difference can be assessed by through-put analysis, by hybridization with allele-specific oligonucleotides (ASO) [43], primer extension [44], oligonucleotide ligation assay (OLA) [45], and invasive cleavage [46]. The theory behind each of these techniques is reviewed in Sabrino *et al*. [47] and Semagn *et al*. [48]. There are numerous SNPs in plant genomes. In a comparison of genomic sequences from indica type rice and japonica type rice, Yu *et al*. [49] demonstrated one SNP in every 170 bp and one in/del every 540 bp. In a genome wide survey of 877 unigenes, SNPs were estimated to be present in every 200 bp in barley [50]. In maize, which is a cross-fertilizing species, Ching *et al*. [51] showed an even higher frequency of SNPs, an average of one polymorphism per 31 bp in non-coding regions and 1 polymorphism per 124 bp in coding regions. Therefore, we can extrapolate that the frequency of the SNPs can range from approximately one per 30 bp to one per 500 bp in other plant species. Although these new generation marker systems are powerful tools in linkage disequilibrium analysis, germplasm assay by haplotyping, QTL (quantitative trait loci) analysis and a few others [52,53], they are only amenable to use in those species for which extensive nucleotide sequence information is available in major crops. Complete genome sequences for an increasing number of plant species are being enlisted each year with rapid technical advancements in DNA sequencing strategies. As a result, molecular marker systems are being utilized more frequently in plant genomics and plant genetics. Thus, we restrict our discussion on the SSR applications to minor crop species in germplasm activities. Table 1 compares the different marker techniques that are employed in plant genome analysis for a variety of criteria.

**Table 1:** Comparison of widely using molecular marker systems for plant genome analyses.

	Isozyme	RFLP	RAPD	AFLP	SSR	SNP
Abundance	Low	Medium	Very high	Very high	High	Very high
Types of polymorphism	Amino acid change in polypeptide	Single base change, insertion, deletion, inversion	Single base change, insertion, deletion, inversion	Single base change, insertion, deletion, inversion	Repeat length variation	Single base change
DNA quality	-	High	Medium	High	Medium	Medium
DNA sequence information	-	Not required	Not required	Not required	Required	Required
Level of polymorphism	Low	Medium	High	High	High	High
Inheritance	Co-dominance	Co-dominance	Dominance	Dominance	Co-dominance	Co-dominance
Reproducibility	Medium	High	Low	Medium	High	High
Technical complexity	Medium	High	Low	Medium	Low	Medium
Developmental cost	Medium	High	Low	Low	High in start	High
Species Transferribility	High	Medium	High	High	Medium	Low
Automation	Low	Low	Medium	Medium	High	High

Note: Data on the RFLP, RAPD, AFLP and SSR were modified from Semagn *et al*. [48].

## What Are SSRs?

In the 1960s, simple repeats that are scattered throughout eukaryotic genomes, were identified in density gradient centrifugations of randomly sheared genomic DNAs by way of a ‘satellite peak’ [54]. Isolation and sequencing of these satellite DNAs revealed repeat motifs of variable length from just a single base to thousands of bases; a typical satellite DNA is a centromeric sequence with a 100 bp repeat [55]. Subsequently, satellites of 10–30 bp repeat motifs, termed minisatellites, were isolated in mammals [18]. Finally, satellites with even shorter repeat motifs, called microsatellites, were isolated. In 1982, Hamada and his colleagues showed the existence of dinucleotide repeats of poly (C-A) and poly (G-T) in diverse eukaryotic genomes [56]. Since the repeat motifs in microsatellites are as short as 1–6 bases long, microsatellites are referred to as simple sequence repeats or simply, SSRs. Subsequent studies by Tautz and Renz [57] confirmed that SSRs, originally designated as STRs (short tandem repeats), were found to be informative, and abundantly present in human, fruit fly, sea urchin, protozoan, and yeast genomes. Furthermore, Weber and May [58] demonstrated that SSR polymorphisms (SSRPs) could be easily detected by PCR, using two flanking primers, which prompted the development of SSRs in various mammalian species and their subsequent assignment to specific chromosomes [59,60,61]. In plants, the presence of SSRs was first demonstrated by the hybridization of oligonucleotide probes of poly (G-T) and poly (A-G) on the phage libraries of tropical tree genomes [62]. A search of published DNA sequences reveals that SSRs are also highly abundant in diverse plant genomes [64,65]. SSRs have therefore become the preferred molecular marker system for analysis in plant genetics and ecology.

**Figure 1:**
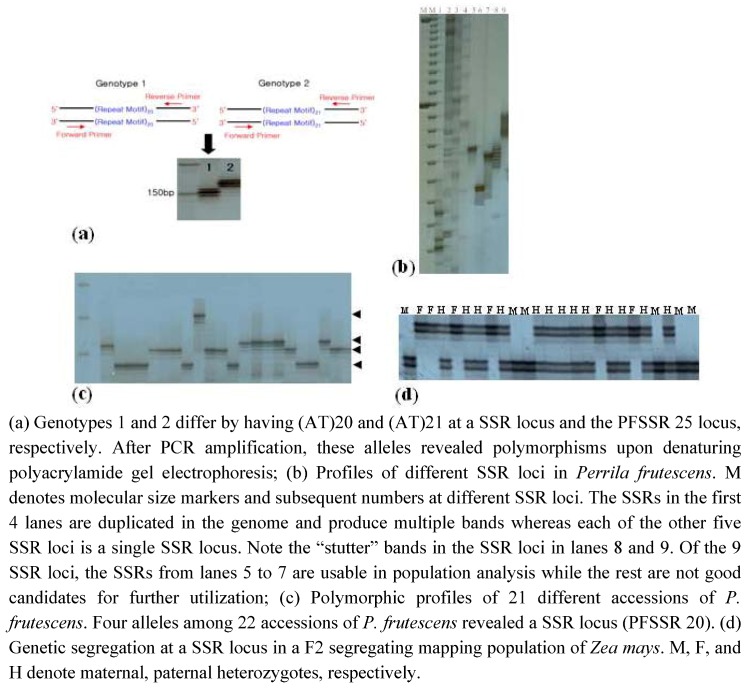
SSR amplification profiles.

The criteria for identifying SSRs differ, depending on the scientists who isolate and characterize them. While some scientists do not recognize single nucleotide repeats as SSRs, some consider the number of repeat units and others consider length of the repeat motifs [63,65,66,67,68,69] as criteria for defining SSRs. Akkaya *et al*. [63] isolated SSRs having more than 4 repeat units of di-, tri-, and tetranucleotide motifs in their analysis with soybean. Temnykh *et al*. [67] isolated 13,989 SSRs with di-, tri-, tetra-nucleotide motifs from 47 Mb of BAC-end sequences of rice, and categorized them into two classes; class 1 - hypervariable markers with motif length ≥ 20 bp, and class 2 - potentially variable markers with a repeat length ≥ 12 bp < 20 bp. However, the issue of repeat motifs is sometimes obscured by slightly imperfect sequences in repeat motif and by composite SSRs having more than one repeat motif. Therefore, describing SSR occurrence in can be specified in each genome sample. In our investigations with minor crop species, we define SSRs as DNA sequences in which a repeat motif length is over 20 bp with lower than 20% sequence imperfection in the repeat motif. Figure 1 describes the protocol for PCR amplification of SSRs with an example of a SSR profile in a population of a *Perrilla* species.

## How Did SSRPs Arise?

**Figure 2:**
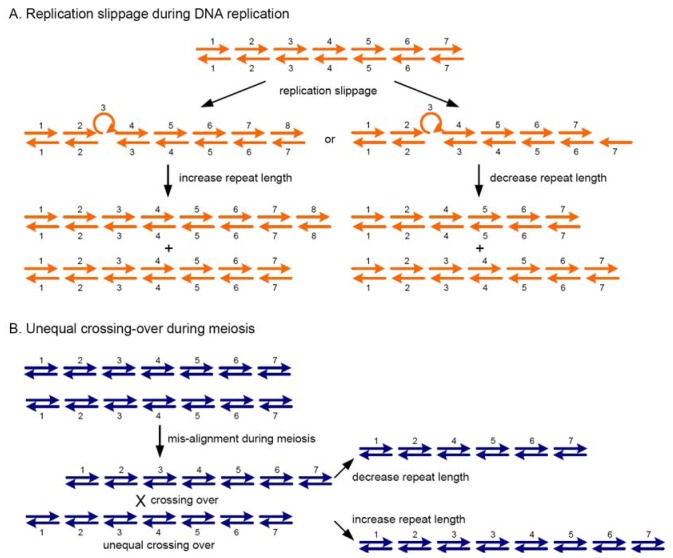
The mechanisms of unequal crossing-over and replication slippage for producing SSRPs.

SSRPs are mostly length variations by the different number of repeat units. It is not certain how these tandem repeats arose in genomes. Initially, it was thought that the occurrence of unequal crossing-over between repeat units during meiosis accounted for the length variation in minisatellites (SSRs) [70] while DNA replication slippage was responsible for the variation in microsatellites [71]. However, several studies have demonstrated that both types of repeats can be derived by either of the two mechanisms [72]. DNA replication slippage can also occur during *in vitro* amplification of the SSRs to produce ‘stutter bands’, which often obscure non-parental SSR bands in genetically segregating populations [73]. Figure 2 illustrates the mechanisms for producing SSRPs through unequal crossing-over during meiosis and slippage during DNA replication. 

**Figure 3:**
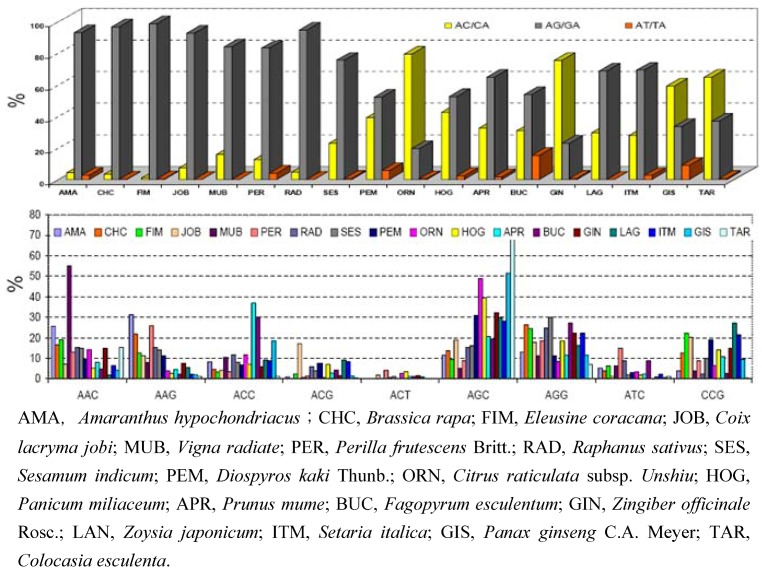
Frequencies of different classes of di-and tri-nucleotide repeat motifs in 18 underutilized crop species.

Both unequal crossing over and DNA slippage can either increase or decrease the number of repeat units in genomes. The number of repeat units ranges from 2 to 40, with extreme exceptions being as many as several hundred, as is the case with the Huntington's disease and fragile-X loci in humans. Is there a bias for specific repeat units in the different genomes? Yes, there appears to be a bias for different repeat motifs in different species, as shown in Figure 3. However, it is not clear why different species harbour different SSR motifs preferentially. Are SSRs with a higher number of repeats more polymorphic than those with a lower number of repeats? Generally, SSRs with longer repeats are more polymorphic than those of shorter ones [73].

## How Do We Develop SSRs?

A variety of protocols have been employed to develop SSRs. There are several excellent reviews on the strategies for the isolation of microsatellites [74,75]. Only a fraction of the original clones from a successful isolation of SSR markers will be amenable to PCR amplification, since the clones containing SSR motifs with primer sequences are reduced at each step [75]. Therefore, optimum protocols are necessary for the efficient isolation of SSR markers in a given species.

**Figure 4:**
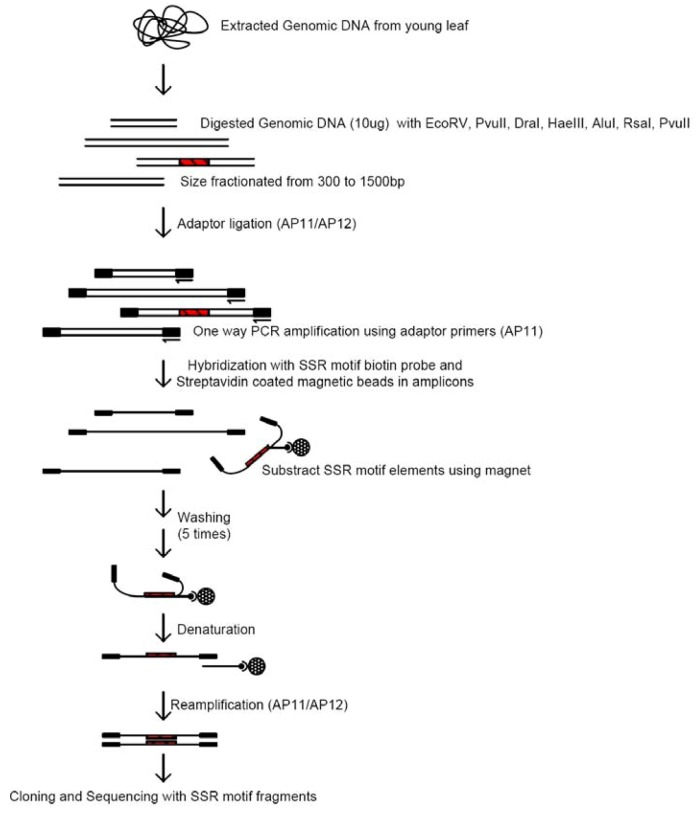
A general protocol for developing SSR markers with a SSR-enrichment step.

Traditionally, SSRs have been isolated from partial genomic libraries containing small size inserts by colony hybridization with probes that contain SSR sequence motifs [62,76,77]. However, this technique has been inefficient in most cases of species having large genomes, with the frequencies of colonies containing SSR motifs being relatively low. A SSR-enrichment step, prior to the library construction, was incorporated into the protocol to circumvent this problem. The SSR-enriched clones can then be employed by direct sequencing [78,79,80,81,82] or by incorporating one more step of colony hybridization with end-labelled SSR probes [83,84,85]. Figure 4 describes the general protocol for developing SSR markers with an SSR-enrichment step. 

Another approach for the isolation of SSRs involves a computational search of the genome databases. Weber and May [58] searched (CA)n SSRs in a human genome sequence database and demonstrated the abundance of the (CA)n SSRs in the human genome. In plants, the first report of (CA)n repeats in soybean sequences was the one from a computer search of the GenBank databases [63]. Around the same time, Morgante and Olivieri [64] showed an abundance of AT repeats in 30 different plant genomes and demonstrated that the repeat number of variations by PCR amplification were highly informative in genome analyses. In 1994, Wang *et al*. [65] surveyed mono-, di-, tri- and tetranucleotide repeats in plant sequences in EMBL and GenBank. They found that mono-, di-, and tetra-nucleotide repeats were all present in non-coding regions, but that 57% of the tri-nucleotide repeats, containing G-C base pairs, resided in the coding region. The high occurrence of tri-nucleotide repeats in coding region was attributed to the other types being eliminated from the coding region because of their ability to cause frame-shift mutations. With the wealth of sequence information of ESTs, fully characterized genes, and full-length cDNAs, computer searches are particularly useful in developing genic SSR markers [68,69,86]. There are a number of web-based SSR search softwares such as: MISA (MIcroSatellite, http://pgrc.ipk-gatersleben.de/misa), CUGssr (http://www.genome. clemson.edu/projects/ssr), Sputnik (http://abajian.nert/sputnik/index.html), and SSRSEARCH (ftp://ftp.gramene.org/pub/gramene/software/scripts/ssr.pl). However, with species that have scant information on their genomic sequences, as is the case with most small or minor crop species, a computer search would not be useful in developing SSR markers. Library construction with SSR-enriched sequences would be a plausible way to develop SSR markers in these neglected minor crop species. 

## Advantages of SSR Analysis

SSR markers have many advantages over the other marker systems. The first advantage is their high reproducibility, which would be the most important in genetic analysis. While reproducibility of the SSR profile is as robust as it is with RFLPs, experimental procedures for SSR analysis are much simpler and require only a small amount of template DNA. Since SSR analysis does not require restriction with enzymes, it can reproduce the same profiles regardless of the state of the template DNA. It also does not require template DNA to be ultra pure, which is a requirement in AFLP analysis since contaminated or impure DNA is often recalcitrant in restriction enzyme digestions to produce nonspecific spurious bands. This is a real benefit when one is dealing with specimens that are dry, contaminated, mummified or even in fossilized form in the wild [87,88].

The second advantage of the SSR marker system is the polymorphic genetic information contents. The hyper-variable nature of SSRs produces very high allelic variations even among very closely related varieties. A literature survey showed that the number of alleles varied from 1 to 37 with diversity indices of 0.29–0.95 in major crop species [89]. The level of genetic variation detected by SSRPs analysis was almost two times higher than that detected by RFLPs, with 61 soybean lines [90]. In a comparative study of the utility of RFLP, RAPD, AFLP, and SSR marker systems for germplasm analysis, SSRs showed the highest expected heterozygosity, while AFLPs had the highest effective multiplex ratio [89].

The third advantage has to do with the co-dominant nature of SSR polymorphisms. Although homoplasious bands can be misleading in scoring SSR profiles, the SSR bands produced from the same set of primers are intuitively orthologous (a more detailed discussion of homoplasy is provided in the ensuing section). The multiple bands generated from RAPD and AFLP analyses do not permit their designation as allelic or orthologous bands until they are converted into STS markers after sequencing. The co-dominant nature of SSRPs is suitable for genetical analysis in segregating F2 populations or parentage analysis in hybrids [91,92].

The fourth advantage of the SSR marker system is their abundance and distribution in genomes. As more and more genomic sequences are being identified in various eukaryotic species, it is becoming increasingly evident that SSRs are truly abundant in almost all species, and are well distributed throughout their genomes [65,68,93]. Genetic analysis is often frustrated by the fact that large numbers of anonymous RAPD or AFLP markers are clustered in specific locations of chromosomes or linkage maps [94,95]. In search of SSRs longer than 12 bp in a 57.8 Mb, publicly available rice (*Oryza sativa* L.) sequence, Temnykh *et al*. [67] showed that many kinds of SSRs are present every 16 kb. In another survey of SSRs in different eukaryotic genomes, Tóth *et al*. [93] reported that coding and non-coding regions differed significantly in SSR distribution, and characteristic differences also existed between inter-genic regions and introns in eukaryotes from yeasts to mammals to plants. Like the early findings in plants by Wang *et al*. [65], tri- or hexa-nucleotide SSRs were predominantly present in coding regions, in the study by Tóth *et al*. [93].

A fifth advantage of the SSR marker system is that SSRs are preferentially associated with non-repetitive DNA [68,86,96]. Genomic sites of SSR markers, derived from genomic libraries, fall into either the transcribed region (genic SSRs) or the non-transcribed region (genomic SSRs). The SSRs, derived from ESTs or cDNAs, are mostly genic SSRs, which have the potential for application in such areas as gene function characterization [97], association analysis for gene tagging [98,99,100], and QTL analysis [101,102,103]. However, this last advantage can only be applied to those species with large amounts of EST or cDNA sequences that are freely accessible to public.

## Problems with SSRPs

Although analysis with SSR markers has many merits compared to other marker systems, there are a few inherent problems associated with this system. Since SSR sequences can cause replication slippage *in vitro*, SSR polymorphisms are sometimes derived from slippage during polymerase chain reactions [73,104]. Slippage during PCR produces ‘stutter bands’ that differ in size from the main product by multiples of the length of repeat unit [104,105,106]. The stuttering produces many ladder bands in polyacrylamide gel separation, and leads to quasi-scoring, if there are no prominent bands among the ladders. Stuttering also generates ambiguity in SSRs with long stretches of a short repeat unit (1-2 bp) since Taq polymerase slippage increases with the number of repeat units, and is inversely correlated with the length of the repeat unit [107]. By rating the quality of the SSR-PCR products on a scale of 1 to 5 (good to poor), it is suggested that only those SSR primers with a rating of 1 and 2 be used in genomic analyses [106,108].

Homoplasy is an evolutionary term that is used to describe the observation that a character present in two species is not derived from a common ancestry but rather, the similarity is a result of convergence, parallelism or reversion. In the case of genetic markers, homoplasy is a phenomenon wherein different copies of a locus are identical in state despite not being identical by descent [109]. In SSR analysis, homoplasy can occur if two bands are similar in size but not identical in sequence. Table 2 illustrates four electromorphs, each 214 bp in size, from four different *Apis* species [110]. Two sets of identical electromorphs can be identified; one from *A. millifera* and *A. lingustica* and the other from *A. scutellata* and *A. capensis*, respectively. However, the sequences were slightly different between sets by size homoplasy. The homoplasy can be confirmed by nucleotide sequencing or electrophoresis by single strand conformation polymorphism [111]. Homoplasy can lead to an underestimation of the actual divergence between populations [110,112,113]. However, SSR electromorph homoplasy does not present a significant problem in population genetics analyses since large amounts of variability at SSR loci are often compensated for by their homoplasious evolution, as shown in a computer simulation analysis with a large data set [109]. SSRP detects size variation by the differences in the number of repeat units, which arose by replication slippage or unequal crossing over. If a SSR with 5 repeat units is extended to a SSR with 6 repeat units, the latter can reduce its repeat unit number to 5 by a single ‘back-mutation’. If this happens, SSRP analysis cannot differentiate between the original SSR locus with 5 repeat units and the back mutated SSR locus with 5 repeat units, even though two steps of mutation were involved. This type of homoplasy cannot be checked, and may also lead to an underestimation of the genetic diversity of a population [114,115].

**Table 2:** SSR size homoplasy at the locus A113 in different subspecies of *Apis mellifera*.

Sub-species	Electromorph (size in bp)	Core sequence
*Apis mellifera*	214	(TC)_2_C(TC)_2_TT(TC)_4_TT(TC)_2_TT(TC)_9_GTTTCG(TC)_2_
*Apis ligustica*	214	(TC)_2_C(TC)_2_TT(TC)_4_TT(TC)_2_TT(TC)_9_GTTTCG(TC)_2_
*Apis scutellata*	214	(TC)_2_C(CT)_2_TT(TC)_5_TT(TC)_11_GTTTCG(TC)_2_
*Apis capensis*	214	(TC)_2_C(CT)_2_TT(TC)_5_TT(TC)_11_GTTTCG(TC)_2_

Note: Data from Estoup *et al*. [110] with modification.

Often, in SSR analyses of a large number of samples from diverse germplasms, a few samples fail to produce PCR products [116,117,118,119]. This is a source of frustration to experimenters because they cannot determine whether the absence of PCR products represents true null alleles of the SSR locus or is due to a failure of the PCR reaction. Null SSR alleles represent the phenomena that do not give a PCR product by mutations [119,120,121,122]. Any individuals do not show amplification any alleles repeatedly while amplify normally other loci, null alleles may be present in this locus. If re-extracted DNA from the individual fails to show an amplification product, it is highly likely that the individual is homozygous for that allele. SSRs derived from ESTs or cDNA often fail to produce PCR products if one or both primer binding sites happen to be on the splice sites. Presence of large introns or primers designed from chimeric cDNA will not produce successful PCR products. Sometimes, primers designed from poor quality sequence information generate unsatisfactory results. Although the low rate of null alleles may be ignored in most population analyses, they may pose some complexity in parentage analysis as shown by Dakin and Avis [120]. It is recommended that rigorous primer selections be done for consistent amplification across all the samples before large scale analyses are conducted.

## Cross-Species Applications

If SSRs are isolated for which primers can be designed, there is no doubt that the SSR marker system has many advantages over other marker systems. If the sequence information is insufficient to develop SSR markers, it may be advantageous to utilize primer sequences identified for one species in the analysis of other closely related species, given the high cost of developing useful SSR markers. SSRs from non-coding regions were not successful in cross-species amplifications due to the sequence variation surrounding SSR motifs, whereas SSRs from coding regions were successful in a wide range of species. In the most extensive cases, 17 SSRs were able to amplify across fish, which diverged about 470 mya (million years ago) [123]; six SSRs isolated from marine turtles amplified freshwater turtles, which diverged about 300 mya [124]. Peakall *et al*. [125] demonstrated that although 31% of soybean SSR loci were transferable to other legume species, the useful transferability was restricted to congeners. In grass species, Chen *et al*. [126] selected 11 SSR markers from *Oryza sativa* with the following criteria: (i) high allelic variation in 13 *O. sativa* cultivars, (ii) a variety of perfect and compound SSR motifs, and (iii) minimal stutter bands. The 11 SSR loci were all amplifiable among the *Oryza* species having the same A genome, whereas 73% (8/11) of primers amplified *Oryza* species having the other genomes B and C, and 27% (3/11) amplified species in other genera. Transferability of EST-SSR markers is high. Gupta *et al*. [119] demonstrated that 43 of 78 EST-SSR markers exhibited transferability from *Triticum* to *Hordeum*, indicating that the sequences flanking SSR motifs were conserved not only within a single genus but also between related genera in the Poaceae family. However, SSR markers derived from a genomic SSR-enriched library showed poor cross-species amplification between species from a different genus. Only two of eleven SSR markers from an enrichment library of *Swietenia humilis* showed amplification across the Meliaceae family [127]. Regardless, it can be concluded that SSR primers from a SSR-enrichment library are still useful in the analysis of species within a genus. Recently, we isolated 12 SSR loci from *Amaranthus hypochoriacus* and were able to demonstrate cross-amplification of these SSR markers to 18 other wild species in the genus *Amaranthus* [82]. Similar results were obtained with the SSRs isolated from the common buckwheat (*Fagopyrum esculentum*) in cross-species amplifications with other species in the genus *Fagopyrum* [128].

One should be cautious in using transferable SSR markers for assessing species relationships since the maintenance of allele sizes among species is complex. The complexity of the mutation process in SSRs as well as size homoplasy may complicate the interpretation of SSR variations. Size homoplasy was frequently detected among cross-species amplified SSR markers between species. In the study of cross-species amplifications in *Oryza* species, Chen *et al*. [126] demonstrated that mutations occurred not only in the repeat units but also in the flanking regions to show allele size homoplasy as well as cryptic alleles. Similar results have been reported in studies with *Pinus* species [129] as well as legume species [125]. Therefore, it is recommended that inferences vis-a-vis species relationships using SSRPs be accompanied by the information underlying sequences. 

## Biological Functions of SSRs

SSRs were generally deemed to be evolutionarily neutral. However, numerous lines of evidence have demonstrated that SSRs are not distributed randomly in the genome [86,93]. It is estimated that 14% of the genes in eukaryotic species contain repeated sequences, approximately three times more than in prokaryotes [130]. Incorporation of repeat sequences in eukaryotic genomes may confer an evolutionary advantage of adaptability to new environments [130,131]. Debates on the functional role(s) of the SSRs on species adaptation and survival have been well documented [132,133]. However, the findings of expansion and contraction of the SSR motifs within genes have encouraged the assignment of a biological role to SSRs. Thus far, the best known cases of SSRs with phenotypic effects are the human loci of Huntington's disease, and fragile-X [134]. SSRs on the UTR regions may also be involved in the regulation of expression of nearby genes as shown by a GT repeat in the Tilapia *prolactin* 1 gene in fish, in response to a salt-challenged environment [135]. Intronic SSRs can regulate gene expression by influencing mRNA splicing or by translocation of mRNA to cytoplasm, as shown by the CCTG repeat in the first intron of the human zinc finger protein 9 (ZNF9), in which an expansion of the repeat causes one intron splicing to fail, thus leading to myotonic dystrophy [136]. Although biological roles for SSRs in plants have not been reported as yet, similar roles are expected for these molecular markers in plant genes. 

If some SSRs are functional and confer an adaptive advantage, are these functional SSRs suitable in the assessment of biodiversity and ecological conservation of endangered species? Most of the molecular markers that have been utilized in population genetics have not undergone selection, and therefore have been essentially neutral. In neutral theory, the frequency of alleles is determined by purely stochastic processes [137]. In conservation biology, neutral molecular markers may be useful in providing fundamental information about the types of mating in a population, gene flow, and the population history of a species [138]. However, there was a large discrepancy between genetic divergence as measured by neutral RAPD markers and that measured by quantitative genetic traits of the monkey puzzle tree (*Araucaria araucaucana*), a vulnerable tree endemic to southern South America [139]. Tienderen *et al*. [140] contend that gene-targeted functional markers can contribute to ex-situ management of genetic resources, studies on ecological diversity, and conservation of endangered species. Holderegger *et al*. [141] proffer a theory on the adaptive versus neutral diversity for landscape genetics in which the diversity measured by neutral markers is well suited for the study of processes of gene flow within landscapes, whereas diversity assessed by quantitative genetic experiments using functional markers is best suited for measuring the evolutionary or adaptive potential of a population or species. They concluded that ecologists must recognize these differences between neutral and adaptive genetic variation when interpreting the results of landscape genetic studies. However, it should be remembered that variation in functional genes might reflect the past influence of selection, which can be variable in each gene and can affect the profiles of variation from the history, migration, and drift [141]. While genomic SSR markers are mostly neutral, genic SSRs from EST's or cDNAs may retain some adaptive roles. This duality in selection and adaptation ascribes another advantage to the utilization of SSRs in characterizing the genetic diversity of the resources that are preserved in different germplasm institutes.

## Germplasm Collections and Development of SSRs in Minor Crops

Minor crops are also referred to as ‘orphan crops’ or ‘underutilized crops’ because of their lack of global cultivation and utilization [142]. Compared to major staples or other economic crops, these minor crops have often been neglected and are therefore on the verge of extinction in some cases. Human activities in the wild have also accelerated the genetic erosion and extinction of these vulnerable plants which are listed in the website of The International Centre of Underutilized Crops (http://www.icuc-iwmi.org/). Albeit late, accessions of these neglected minor crops in gene banks and germplasm institutes have been collected worldwide. However, introduction of the accessions of these minor crops without their characterization, limits the maximum preservation of their genetic diversity [143]. The concept of ‘core collection’ was introduced to counter the problems faced in the management of germplasm (1-3). So, the determination of entries should be done to meet the requirement of least number of accessions with highest diversity of selected subset. At the onset, a core collection was simply defined as a subset of accessions with a good approximation of the genetic diversity of a crop species and its wild relatives from the existing collections. Until the ‘core collection’ concept was established in early 1980s, germplasm was collected from as many accessions or species as was possible; core collection represents genetic diversity with a minimum of repetitiveness in a crop species and its relatives [1,2,3]. The assessment of genetic diversity of introductions (accessions) is now a pivotal strategy for their successful and efficient preservation, in situ as well as ex situ [140]. SSRPs are the most suitable markers for the genetic assessment of germplasms because of their hypervariability, attributable to allelic variations [128,144,145]. In a study of 192 accessions of *Medicago trunculata* with five SSR markers, Ellwood *et al*. [144] showed that the core collection was highly diverse; over 90% of the genotypes, with an average of 25 SSR alleles per locus, defined a subset of accessions (n = 61) that will maximize the diversity. Similarly, Zhao *et al*. [145] studied the genetic diversity and population structure of a selected core of garlic accessions with 8 SSR primers; 95 accessions from 613 garlic entries obtained from 36 different countries captured all the alleles in the entire collection of accessions by the model-based heuristic approach [146]. This heuristic method implements the determination of core entries with least number of accessions and 100% coverage of diversity of entire collection in a selected core sub-set, which is completely different from the conventional methods including the stratification and a previous M-strategy. For a similar assessment of Korean Germplasm collections, SSR motifs of various minor crop species have been isolated to aid in efficient germplasm management [147]. Characterization of these collections is currently underway with the SSRs that have been specifically developed for the purpose. 

## Concluding Remarks

Preservation of genetic diversity is important at both the molecular and species levels, if one generation is to pass on its legacy of intact diverse forms of life to the next generation. Although collections of accessions of neglected or unintended plants or crops are being carried out world-wide in germplasm institutes, a recurrent problem, apart from limited space and other resources, is the redundancy of the introductions, thus necessitating their pre-screening before being placed in the depository. Redundancy screening for molecular diversity through the use of molecular markers is very efficient, and is being widely used. Of the many molecular systems that are available, SSRP is the method of choice because it can also be applied to core collections of species. However, it cannot be directly applied to minor or neglected orphan crops because of the requirement of DNA sequence information for designing primers for PCR. Since the set-up costs for SSR development in new species are exorbitant, it is recommended that DNA sequence information be borrowed from related species for cross-species SSR amplification. In so doing, researchers should be aware that some homoplasious SSR bands can underestimate the molecular diversity. Regardless, SSRPs have more advantages than other molecular marker techniques. With more and more DNA information sequences becoming available through ESTs or whole genome sequencing, the number of available SSR markers is also increasing. Efficient SSR-enrichment library construction protocols are available with various modifications so that researchers can select the protocols to meet their needs. The characterization of the various germplasm introductions through SSRPs, which is well underway, will be of significant benefit in managing the precious genetic diversity of underutilized species.
